# The D75N and P161S Mutations in the C0-C2 Fragment of cMyBP-C Associated with Hypertrophic Cardiomyopathy Disturb the Thin Filament Activation, Nucleotide Exchange in Myosin, and Actin–Myosin Interaction

**DOI:** 10.3390/ijms252011195

**Published:** 2024-10-18

**Authors:** Anastasia M. Kochurova, Evgenia A. Beldiia, Victoria V. Nefedova, Daria S. Yampolskaya, Natalia A. Koubassova, Sergey Y. Kleymenov, Julia Y. Antonets, Natalia S. Ryabkova, Ivan A. Katrukha, Sergey Y. Bershitsky, Alexander M. Matyushenko, Galina V. Kopylova, Daniil V. Shchepkin

**Affiliations:** 1 Institute of Immunology and Physiology of the Russian Academy of Sciences, 620049 Yekaterinburg, Russiacmybp@mail.ru (D.V.S.); 2 Research Center of Biotechnology of the Russian Academy of Sciences, 119071 Moscow, Russia; 3 Institute of Mechanics, Moscow State University, 119192 Moscow, Russia; 4 Koltzov Institute of Developmental Biology, Russian Academy of Sciences, 119334 Moscow, Russia; 5 Department of Biochemistry, Faculty of Biology, Lomonosov Moscow State University, 119991 Moscow, Russia; 6 HyTest Ltd., 20520 Turku, Finland

**Keywords:** cardiac myosin-binding protein C, thin filament activation, hypertrophic cardiomyopathy mutations, actin–myosin interaction, differential scanning calorimetry, in vitro motility assay

## Abstract

About half of the mutations that lead to hypertrophic cardiomyopathy (HCM) occur in the *MYBPC3* gene. However, the molecular mechanisms of pathogenicity of point mutations in cardiac myosin-binding protein C (cMyBP-C) remain poorly understood. In this study, we examined the effects of the D75N and P161S substitutions in the C0 and C1 domains of cMyBP-C on the structural and functional properties of the C0-C1-m-C2 fragment (C0-C2). Differential scanning calorimetry revealed that these mutations disorder the tertiary structure of the C0-C2 molecule. Functionally, the D75N mutation reduced the maximum sliding velocity of regulated thin filaments in an in vitro motility assay, while the P161S mutation increased it. Both mutations significantly reduced the calcium sensitivity of the actin–myosin interaction and impaired thin filament activation by cross-bridges. D75N and P161S C0-C2 fragments substantially decreased the sliding velocity of the F-actin-tropomyosin filament. ADP dose-dependently reduced filament sliding velocity in the presence of WT and P161S fragments, but the velocity remained unchanged with the D75N fragment. We suppose that the D75N mutation alters nucleotide exchange kinetics by decreasing ADP affinity to the ATPase pocket and slowing the myosin cycle. Our molecular dynamics simulations mean that the D75N mutation affects myosin S1 function. Both mutations impair cardiac contractility by disrupting thin filament activation. The results offer new insights into the HCM pathogenesis caused by missense mutations in N-terminal domains of cMyBP-C, highlighting the distinct effects of D75N and P161S mutations on cardiac contractile function.

## 1. Introduction

Hypertrophic cardiomyopathy (HCM) is a hereditary heart disease characterized by a thickening of the ventricular wall, mainly the left one, disruption of the cardiomyocyte arrangement and interstitial fibrosis [1], hypercontractility, and diastolic dysfunction [2,3]. Studies on multicellular preparations of human myocardium and experimental animal models have shown that HCM was associated with raised power output [4], acceleration of myosin cross-bridge cycling [5], and increased Ca^2+^ sensitivity of thin filaments [6,7]. About 50% of mutations leading to hypertrophic cardiomyopathy occur in the *MYBPC3* gene, which encodes cardiac myosin-binding protein C (cMyBP-C) [8,9,10].

cMyBP-C takes part in determining characteristics of cardiac contraction by regulating actin–myosin interaction. It affects the balance of the rate of contraction and relaxation in the myocardium and is essential for maintaining the high level of myocardial stiffness, enabling the heart to effectively eject blood into the aorta [11,12,13,14]. Additionally, cMyBP-C is vital for coupling increased late-systolic ventricular afterload with delayed relaxation [11]. It also affects the length-dependent characteristics of myocardial contraction, which determine the force of contraction and the filling of the left ventricle, contributing, along with titin, to the implementation of the Frank–Starling law that links systolic ventricular pressure and stroke volume to end-diastolic volume [12,15]. At the molecular level, cMyBP-C influences cross-bridge cycling kinetics [16,17,18,19] and the activation of thin filaments in the cardiomyocyte sarcomere [19,20,21,22,23].

The cMyBP-C is a 141 kDa multidomain protein composed of eight immunoglobulin and three fibronectin domains denoted C0-C10. Between the C1 and C2 domains lies an *m*-domain with phosphorylation sites [24,25,26]. The N-terminal part of cMyBP-C interacts with myosin subfragments 1 and 2 (S1 and S2), actin, and tropomyosin [27,28,29,30,31,32,33,34,35]. Domains C5-C10 are aligned longitudinally along the thick filament backbone, where they interact with titin and coiled-coil tails of cardiac myosin [27,29]. Cryoelectron microscopy and thermophoresis suggest that the central domains of cMyBP-C can interact with the S1 and S2 of cardiac myosin [36,37]. The linker region between the C4 and C5 domains allows the protein to bend at a right angle, directing its N-terminal part toward the thin filament [27,36,38].

The C1-m-C2 fragment binds myosin S2, and the *m*-domain phosphorylation causes the dissociation of this fragment, which influences the number of myosin heads that can interact with the F-actin [25,39,40,41,42]. By binding to actin, the C0-C1-m-C2 fragment activates the thin filament, shifting tropomyosin from a blocked or closed position to an open one, thus promoting the interaction of myosin with actin [20,21,22,23]. cMyBP-C increases the sensitivity of the *p*Ca-force dependence of isolated myocardial preparations [16,43,44] and decreases the velocity of the thin filament sliding over myosin in the in vitro motility assay [17,18,19,22,23]. In a transgenic mouse model lacking the N-terminal C0-C1f domains, these domains were shown to be involved in calcium regulation of and influence cross-bridge detachment and tension redevelopment rates [45,46].

The role of cMyBP-C mutations in HCM development at the molecular level remains poorly understood. The most extensively studied mutations are those that result in truncated protein forms. Most pathogenic mutations of cMyBP-C cause frameshifts and premature stop codons, leading to the production of truncated forms and a decrease in the overall level of cMyBP-C (haploinsufficiency) [47,48,49]. The latter increases the rate of cross-bridge cycling, contributing to diastolic dysfunction and hypertrophy of the left ventricular myocardium [47].

The molecular mechanisms of point mutations in cMyBP-C are less understood and are often classified as likely pathogenic or of uncertain significance in the ClinVar database. Some point mutations in the central domains disrupt domain stability [50,51] and impair cardiomyocyte contractility [52], while others affect actomyosin ATPase [53]. Point mutations in the N-terminal domains of cMyBP-C have a complex impact on the actin–myosin interaction, as these domains are essential for this interaction and the activation of the thin filament.

Mutations in the C-terminal subdomain, composed of three connected α-helical segments arranged in a trihelix bundle (THB) region of the *m*-domain, have been well studied. For instance, the L352P and E334K mutations in human cMyBP-C (corresponding to L348P and E330K in mice) linked to HCM have been investigated in biochemical and physiological experiments [54,55]. In a transgenic mouse model, the L348P mutation increases systole duration and slows diastole and the contraction rate of cardiomyocytes, whereas the E330K mutation has the opposite effect [54]. The impact of these mutations varies depending on whether human or mouse protein was used and the experimental approaches. Biochemical experiments revealed that the L352P mutation enhances the cMyBP-C binding to actin [55,56,57]. Electron microscopy showed that it shifts tropomyosin from a blocked to a closed state, prolonging thin filament activation [58]. The E334K mutation does not affect cMyBP-C binding to actin, but the E330K mutation reduces actin affinity [56,57]. Adding the C1-m-C2 fragment with the L348P mutation to skinned mouse trabeculae resulted in greater force activation at low calcium levels compared to the wild-type fragment [55]. However, in a transgenic mouse model, the L348P mutation did not affect the calcium sensitivity of the *p*Ca-force dependence [54].

The Y237S mutation in human cMyBP-C (corresponding to Y235S in mice) located in the C1 domain has been studied in a mouse model and in silico. Skinned myocardial preparations from transgenic mice with this mutation were hypercontractile, displaying accelerated cross-bridge kinetics and increased Ca^2+^ sensitivity of force generation [59]. Molecular dynamics (MD) simulations revealed that the Y235S mutation induced changes in crucial intramolecular interactions, surface conformations, and electrostatic potential of the C1 domain [59].

Since the C0 and C1 domains of cMyBPC contain interaction sites with thick and thin filament proteins and play a role in thin filament activation, mutations in these domains may have significant functional consequences. We investigated the effects of substitutions D75N [60] and P161S [61,62], which involve conservative residues in the C0 and C1 domains, reported as pathogenic/uncertain significance in the ClinVar database, on the structural and functional properties of the C0-C1-m-C2 (C0-C2) fragment. We also discuss possible molecular mechanisms by which these mutations may contribute to the HCM pathogenesis.

## 2. Results

### 2.1. Effects of cMyBP-C Mutations on the Thermal Unfolding of the C0-C2 Fragment Studied with Differential Scanning Calorimetry (DSC)

**Figure 1:**
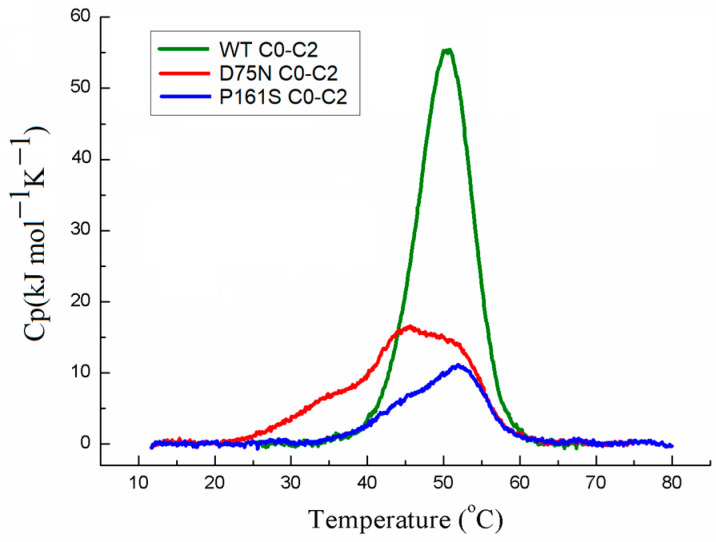
Temperature dependences of excess heat capacity (Cp) monitored by DSC for the WT C0-C2 fragment and C0-C2 fragments with D75N and P161S mutations.

The cMyBP-C mutations D75N and P161S have impacted the structure of the N-terminal C0-C2 fragment of cMyBP-C (Figure 1). The WT C0-C2 protein exhibited a symmetric melting peak with a maximum of 50 °C, and its calorimetric enthalpy (ΔH_cal_) calculated as the area under the excess heat capacity curve was 510.0 kJ/mol. The shape of DSC curves indicates that D75N and P161S mutations altered the character of melting. Also, the thermal unfolding of the D75N C0-C2 mutant began at a lower temperature than the WT protein. Both mutations reduced the calorimetric enthalpy of the C0-C2 thermal unfolding by two–three times compared to the WT protein (Figure 1). The ΔHcal value was estimated at 255.0 kJ/mol for the D75N C0-C2 mutant protein and 155.0 kJ/mol for the P161S mutant, indicating that a large fraction of these C0-C2 mutant molecules were disordered and lacked rigid tertiary structure.

### 2.2. Effects of cMyBP-C Mutations on the Binding of C0-C2 Fragment of cMyBP-C to F-Actin

**Figure 2:**
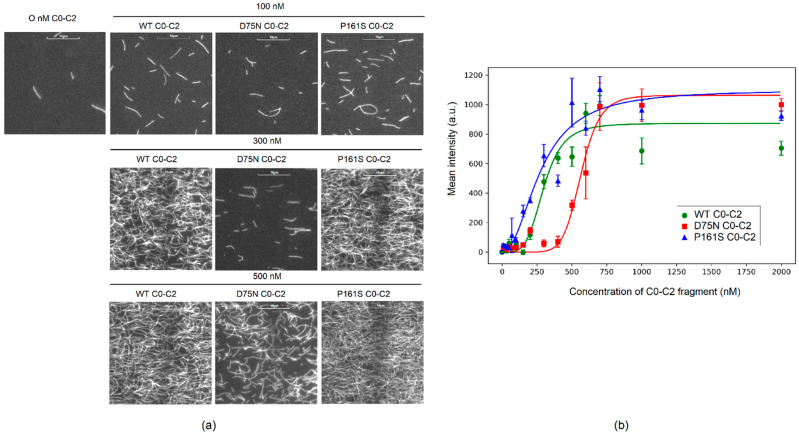
Binding of C0-C2 fragments to F-actin. (**a**) Examples of images of F-actin bound to the flow cell surface at 100 nM, 300 nM, and 500 nM loading concentrations of C0-C2 fragments. (**b**) The dependence of the mean fluorescence intensity in the microscope field of view on the C0-C2 fragment concentration. The intensity was averaged by 10 fields of view in three experiments. Experimental data (mean ± SD) were fitted using the Hill equation corresponding fits shown as lines.

In the absence of C0-C2 fragments, only a few single actin filaments stuck to the BSA-coated surface (Figure 2a). As the concentration of C0-C2 fragments increased, the amount of F-actin in the microscope field of view and the mean fluorescence intensity increased. The D75N C0-C2 fragment exhibited weaker binding to F-actin compared to the WT C0-C2 fragment (Figure 2b). To achieve half the maximum intensity, 299 ± 4 nM of WT C0-C2 fragment, 566 ± 8 nM of D75N C0-C2 fragment, and 277 ± 2 nM of P161S C0-C2 fragment were required.

### 2.3. Effects of cMyBP-C Mutations on the Actin–Myosin Interaction

**Figure 3:**
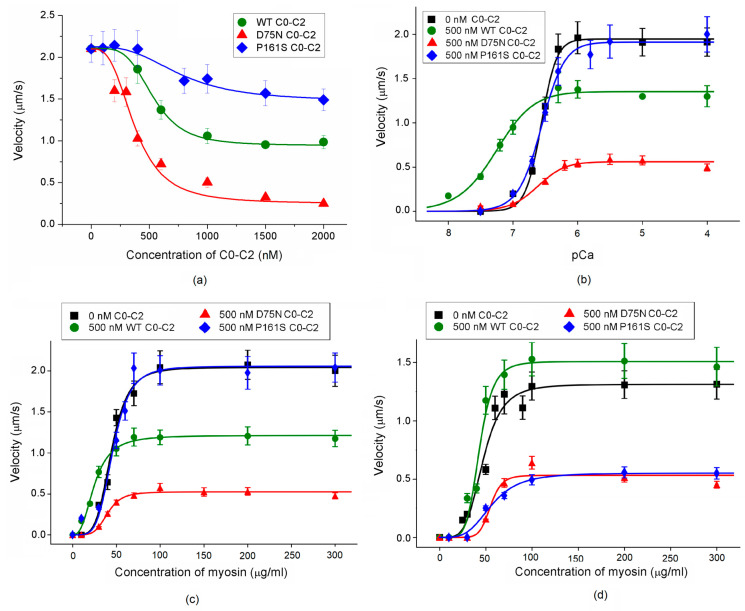
Effects of cMyBP-C mutations in the N-terminal part of cMyBP-C on the actin–myosin interaction. (**a**) Dependence of the sliding velocity of thin filaments over myosin in the in vitro motility assay on the C0-C2 fragment loading concentration at *p*Ca4. (**b**) Calcium dependence of the sliding velocity of thin filaments over myosin. (**c**) Effect of cMyBP-C mutations on the relationship between the thin filament sliding velocity and myosin concentration at *p*Ca4. (**d**) Influence of cMyBP-C mutations on the dependence of the sliding velocity of F-actin–Tpm filaments on myosin concentration. In (**a**), the experimental data (mean ± SD) are fitted by the logistic function. In (**b**–**d**), the data (mean ± SD) are fitted to the Hill equation. The equation parameters are given in Table 1 and Table 2.

We first examined the impact of cMyBP-C mutations on the maximal sliding velocity of thin filaments at *p*Ca4. All studied C0-C2 fragments reduced the velocity, with the D75N fragment causing a greater reduction compared to the WT and P161S fragments (Figure 3a). To achieve a two-fold decrease in the maximal sliding velocity, the following amounts were required: 533 ± 52 nM of the WT fragment; 374 ± 62 nM of the D75N fragment; and 764 ± 96 nM of the P161S fragment. Note that the filament sliding was linear both without fragments and with the addition of 500 nM of any fragment (Figures S1–S4 in Supplementary Materials).

To explore how cMyBP-C mutations affect calcium regulation of the actin–myosin interaction, we analyzed the calcium dependence of the sliding velocity of regulated thin filaments reconstituted from F-actin, troponin (Tn), and tropomyosin (Tpm) on myosin in the in vitro motility assay (Figure 3b, Table 1).

**Table 1:** Characteristics of the calcium regulation of actin–myosin interaction.

C0-C2 Fragment	*V*_max_, µm/s	*p*Ca_50_
0 nM C0-C2	2.0 ± 0.1	6.56 ± 0.01
500 nM WT C0-C2	1.4 ± 0.1 ^#^	7.26 ± 0.01 ^#^
500 nM D75N C0-C2	0.6 ± 0.1 *^#^	6.62 ± 0.01 *
500 nM P161S C0-C2	2.0 ± 0.1 *	6.56 ± 0.01 *

*V*_max_ is the maximal velocity of thin filaments; *p*Ca_50_ (i.e., Ca^2+^-sensitivity) is *p*Ca at which half-maximal velocity is achieved. The * symbol indicates the statistically significant difference between the equation parameters in the presence of the C0-C2 fragments with mutations from those with the WT C0-C2 fragment, *p* < 0.05. The # symbol denotes the statistically significant difference between the equation parameters in the presence of the C0-C2 fragments and those in their absence. Statistical significance was estimated using Mann–Whitney test.

Cooperative mechanisms play a crucial role in the actin–myosin interaction, including cross-bridge–cross-bridge cooperativity [63,64]. Since both mutations in the C0-C2 fragment reduce the calcium sensitivity of the actin–myosin interaction, it is plausible that they weaken the activation of thin filaments by myosin cross-bridges. To investigate this, we analyzed the dependence of the sliding velocity of thin filaments on myosin concentration in the flow cell at saturated calcium concentration (*p*Ca4). For both cMyBP-C mutations, a higher myosin concentration was required to achieve a half-maximal velocity (*c*_50_ at *p*Ca 4), suggesting impaired cross-bridge–cross-bridge cooperativity (Figure 3c).

Given that the C0-C2 fragment interacts with Tpm, we also examined the impact of cMyBP-C mutations on the cross-bridge–cross-bridge cooperativity of myosin interaction with F-actin–Tpm filaments. An addition of 500 nM WT C0-C2 fragment increased the sliding velocity of these filaments without affecting *c*_50_ (F-actin–Tpm) (Figure 3d, Table 2). In contrast, mutations decreased the sliding velocity of F-actin–Tpm filaments and increased *c*_50_ (F-actin–Tpm) (Figure 3d, Table 2). These results indicate that the D75N and P161S mutations in cMyBP-C do indeed affect the activation of thin filaments.

**Table 2:** Effects of the mutations on cross-bridge–cross-bridge cooperativity.

C0-C2 Fragment	*c*_50_ (*p*Ca4), µg/mL	*c*_50_ (F-Actin–Tpm), µg/mL
0 nM C0-C2	44.4 ± 0.7	47.1 ± 1.5
500 nM WT C0-C2	25.0 ± 0.1	43.2 ± 1.0
500 nM D75N C0-C2	40.1 ± 1.2 *	55.1 ± 1.1 *
500 nM P161S C0-C2	46.1 ± 0.1 *	56.4 ± 1.0 *

*c*_50_ (*p*Ca4) is the myosin concentration at which the sliding velocity of thin filaments reaches its half-maximal value at *p*Ca4; *c*_50_ (F-actin–Tpm) is the myosin concentration at which the sliding velocity of F-actin–Tpm filaments is half-maximal. The symbol * indicates the statistically significant difference between the parameters of the equation in the presence of the C0-C2 fragments with mutations from those of the WT C0-C2 fragment, *p* < 0.05. Statistical significance was estimated using Mann–Whitney test.

**Figure 4:**
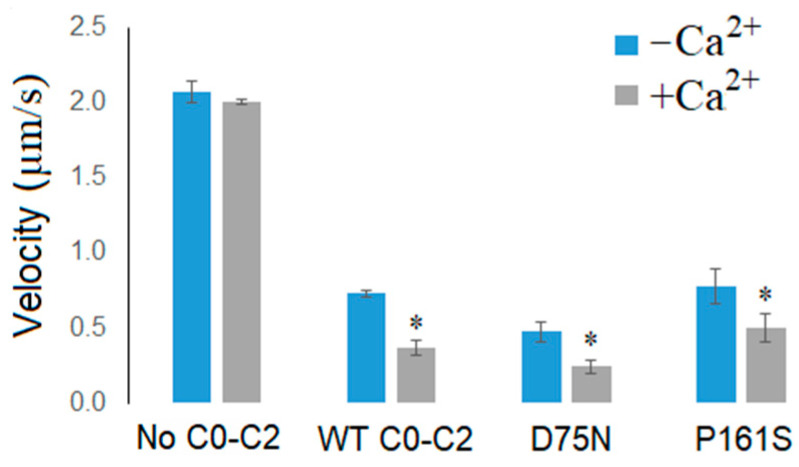
Effect of saturated Ca^2+^ concentration on the sliding velocity of F-actin over myosin in the presence of 500 nM cMyBP-C fragments. The velocity is presented as the mean ± SD. The symbol * indicates the statistically significant difference between the sliding velocity of F-actin at saturating Ca^2+^ concentration (+Ca^2+^) from those without Ca^2+^ (−Ca^2+^), *p* < 0.05. Statistical significance was estimated using the Student’s *t*-test.

Previs et al. [25] demonstrated that the N-terminal domain of cMyBP-C binds Ca^2+^, leading to a reduction in the sliding velocity of F-actin in the in vitro motility assay. We investigated the effects of Ca^2+^ and the C0-C2 fragments of cMyBP-C on the sliding velocity of F-actin. In the absence of the fragments, Ca^2+^ at saturated concentration (*p*Ca 4) did not affect the velocity (Figure 4). The addition of 500 nM WT fragment reduced the velocity by approximately three-fold in the absence of Ca^2+^. With 500 nM WT and D75N fragments, the addition of Ca^2+^ halved the velocity, whereas the presence of the P161S fragment reduced the velocity by 30%.

**Figure 5:**
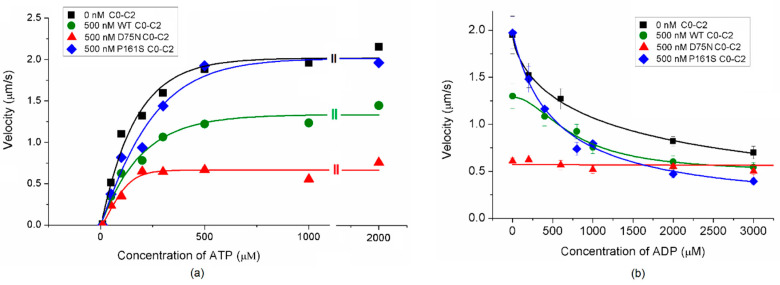
(**a**) Dependence of the sliding velocity of thin filaments on the ATP concentration. The experimental data are fitted to the Hill equation. (**b**) Dependence of the sliding velocity of thin filaments on the ADP concentration. The experimental data (mean ± SD) for 500 nM D75N C0-C2 fragment are fitted by a linear function; experimental data (mean ± SD) for 0 nM WT C0-C2 fragment, 500 nM WT C0-C2 fragment, and 500 nM P161S C0-C2 fragment were fitted to the Hill equation. The values of the ATP and ADP concentration, at which the velocity was half-maximal, are given in Table 3.

Additionally, we examined the effects of cMyBP-C mutations on ATP binding and ADP release by analyzing the dependence of the filament’s velocity on concentrations of ATP and ADP in the in vitro motility assay that allows one to characterize the myosin affinity to these nucleotides indirectly. In the presence of the D75N mutation, the ATP concentration required for the half-maximal sliding velocity of thin filaments (*c*_ATP_) was significantly lower compared to the WT C0-C2 fragment (Figure 5a, Table 3). The *c*_ATP_ value for the P161S fragment was similar to that of WT C0-C2.

ADP dose-dependently decreased the sliding velocity of thin filaments without adding the C0-C2 fragments and in the presence of the WT and P161S fragments (Figure 5b). The results obtained with the D75N mutation are intriguing. The velocity of the filaments with the D75N C0-C2 fragment did not vary on the ADP concentration (Figure 5) and even at 3000 μM ADP, the filaments moved linearly throughout the recordings of the experiment, i.e., over 20 frames (Figure S5 in Supplementary Materials). The ADP concentration that reduced the sliding velocity of thin filaments to half of the maximal (*c*_ADP_) with the WT and P161S fragments did not differ statistically (Figure 5b, Table 3).

### 2.4. Molecular Dynamics (MD) Simulations

**Figure 6:**
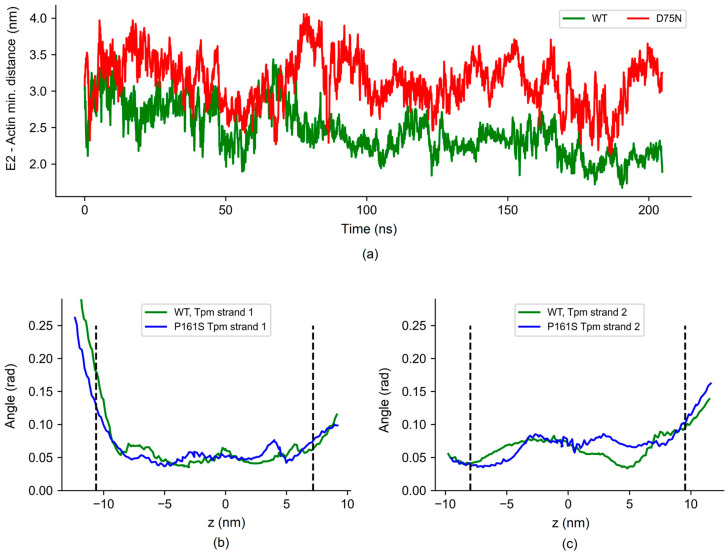
(**a**) The minimal distance between Glu2, first charged N-terminal residue of C0 domain, and actin surface in MD trajectory. (**b**,**c**) Fluctuations of Tpm strands from the actin helix shown as standard deviations of the mean of the azimuthal angles of the residues in two chains of the Tpm strand 1 and 2, respectively, from the actin helix defined by the positions of the K328 residues in the corresponding long pseudo-helical actin strand.

Recent studies resolved the atomic structures of C0 and C1 fragments complexed with actin with two C0 binding modes [20]. We used one of these modes, where the 75th residue was positioned close to the actin surface (PDB ID 6cxj), to investigate the impact of point substitution on the C0–F-actin interactions. The model included five actin monomers and five pairs of C0 and C1 domains. As boundary actin monomers and cMyBP-C domains were more mobile in the MD runs, we used only three central actin monomers for the analysis. The number of hydrogen bonds (*h*-bonds) between the 75th residue and actin decreased to nearly zero with a point mutation (1.2 ± 1.1 for WT vs. 0.0 ± 0.1 for the D75N model), while the total number of *h*-bonds between C0-domain and actin remained nearly constant (5.2 ± 2.4 for WT model vs. 5.4 ± 2.6 for D75N one). However, the disordered N-terminal end of the C0-domain with the D75N substitution showed more distant positions from the actin surface (Figure 6a).

As far as the C1 domain interacts with tropomyosin, we tested the effect of the P161S substitution on this interaction. For this, we simulated the interactions of C1 domains of cMyBP-C with F-actin and Tpm. The atomic structure of C1 cMyBP-C domains with F-actin and Tpm [65] was used to construct the MD model. In this model, Tpm strands with unresolved side chains in the original model [65] were substituted with fully resolved Tpm in the open state [66], and an extra pair of actin monomers with C1-domains of cMyPB-C were added (see Methods). The P161S substitution did not significantly affect the contacts with actin and Tpm. The average number of *h*-bonds between actin and C1-domains, as well as between Tpm and C1-domains, remained unchanged. Additionally, the azimuthal positions of the Tpm strand on the actin helix were similar, and azimuthal fluctuations were comparable for the WT and P161S C1 models (Figure 6b,c).

## 3. Discussion

The molecular mechanism underlying HCM development caused by point mutations in *MYBPC3* remains poorly understood. To shed light on the potential molecular mechanisms of HCM linked to these mutations, we investigated the effect of point mutations of the N-terminal domains on the structure and regulatory function of the C0-C2 fragment of cMyBP-C.

### 3.1. Effects of the Mutations on the Properties of the cMyBP-C C0-C2 Fragment

Our findings reveal that the D75N and P161S mutations located in the C0 and C1 domains significantly impact the stability of the C0-C2 fragment. On the other hand, since only fragments of the residues 1–95 and 151–258 are resolved in the available atomic structures of cMyBP-C (6cxi and 6cxj; [20]), the lack of a link between them does not allow us to confidently judge the dynamics of the N-terminal part of this long molecule as a whole.

The D75N mutation decreases the affinity of the C0-C2 fragment to actin, while the P161S mutation does not affect its binding to actin (Figure 2). MD simulations further suggest that although the D75N mutation causes local destabilization and weakening of the interaction of this region of cMyBP-C with actin, the total number of hydrogen bonds of the C1 and C0 domains with actin remains almost unchanged. Conversely, the P161S substitution does not induce structural disturbances and preserves effective contact of the C1 domain with actin.

The C0 and C1 domains are crucial for cMyBP-C interaction with actin and Tpm and are involved in thin filament activation. Both mutations significantly reduced the calcium sensitivity of the actin–myosin interaction (Figure 3b). This change in calcium regulation may result from mutations suppressing filament activation by cross-bridges (Figure 3c,d). In the in vitro motility assay at saturated calcium concentrations, the D75N mutation reduced the sliding velocity of thin filaments compared to the WT C0-C2 fragment, while the P161S mutation increased it (Figure 3a,b). The difference in the effects of mutations on the filament velocity cannot be solely attributed to their binding to actin and the drag-force mechanism. Despite the D75N mutation significantly decreasing the affinity of the C0-C2 fragment to actin (Figure 2a), it reduced the velocity of thin filaments compared to the WT C0-C2 fragment. Conversely, the P161S fragment, with an affinity to actin similar to that of WT, increases the filament velocity. The impact of cMyBP-C mutations on the thin filament velocity is likely mediated through interaction with Tpm, myosin, or both. cMyBP-C is known to influence the movement of the Tpm strand over the actin surface [20,21,22,23]. The influence was confirmed experimentally with the F-actin–Tpm filament, that is, in the absence of Tn (Figure 3d). The WT fragment slightly increased filament velocity, indicating activation of the F-actin–Tpm filaments, whereas both mutations reduced the velocity by a factor of three (Figure 3d). Additionally, the mutations impaired myosin function by negatively affecting cross-bridge activation.

The complete elimination of the effect of the C0-C2 fragment with the P161S mutation on the thin filament velocity in the presence of Tn (Figure 3b,c) was puzzling. It has been noted that the N-terminal fragment of cMyBP-C interacted with Tpm [20,21] and cTn [67]. These observations suggest that the C0-C2 fragment with the P161S mutation might maintain the open state of the thin filament, which affects these interactions. However, our MD simulations indicate that the P161S mutation does not alter the interaction of the C1-domain with Tpm. Due to the lack of full atomic structures of Tn in the thin filament, we could not include cTn in the modeling. Another possible explanation for the P161S mutation effect could be a difference in the interaction of the P161S fragment with Ca^2+^ compared to WT C0-C2 (Figure 4).

### 3.2. Mechanisms of HCM Pathogenesis

The development of HCM due to mutations in sarcomeric proteins involves increased activation of the thin filament and elevated ATP consumption [68]. Increased calcium sensitivity and thin filament activation lead to stronger contraction and impaired relaxation of the ventricular myocardium. The resultant increase in ATP consumption causes energy deficiency, contractile dysfunction, myocardial hypertrophy [5], and/or diastolic dysfunction [69,70]. cMyBP-C affects thin filament activation and ATP consumption by myosin, thus affecting contractile characteristics.

cMyBP-C inhibits myosin cycling on actin at high Ca^2+^ concentrations and supports their interaction at low Ca^2+^ concentrations, which is crucial for normal systole and diastole duration [11,12,13,14,52]. At the whole-heart level, cMyBP-C slows contraction rates, facilitating efficient blood ejection into the aorta during systole [11,12,13,14,52]. It also regulates systolic kinetics and diastolic relaxation in the intact heart [11,12,13,14,52]. Our data show that the P161S C0-C2 fragment does not inhibit the maximal sliding velocity of filament in the in vitro motility assay, unlike the WT fragment. This suggests that the P161S mutation may lead to a shortened systole duration, potentially contributing to hypertrophic cardiomyopathy, as observed in *MYBPC3* knockout mouse hearts [12,46,71,72].

Energy balance is a critical factor in the onset of myocardial hypertrophy [68]. cMyBP-C participates in modulating ATP/ADP exchange kinetics within the ATPase pocket of cardiac myosin [73,74]. In our study, the D75N mutation caused a three-fold reduction in *c*_ATP_ compared to the WT cMyBP-C (Figure 5a, Table 3), suggesting that this mutation increases the affinity of cardiac myosin to ATP during its interaction with the thin filament. Additionally, ADP reduced the sliding velocity of thin filaments in the absence of the C0-C2 fragment of cMyBP-C and the presence of WT and the P161S fragments (Figure 5b). However, in the case of the D75N mutation, the sliding velocity remained unaffected by ADP in concentrations up to 3 mM (Figure 5b). This observation suggests that the D75N mutation significantly disrupts nucleotide exchange kinetics by decreasing ADP affinity to the ATPase pocket and slowing the myosin cycle.

The mechanisms of point mutations in N-terminal domains are extremely diverse. For example, the L348P mutation in the *m*-domain prolongs systole and leads to diastolic dysfunction, contributing to myocardial hypertrophy [56]. At the molecular level, this mutation affects thin filament activation. The D389V mutation located in the C2 domain disrupts the interaction of cMyPB-C with the myosin S2 fragment, thereby accelerating the kinetics of myosin interaction with actin without altering calcium transitions in the cell [75]. This mutation has been shown to reduce the sliding velocity of actin filaments over myosin in the motility assay, promoting hypercontraction and myocardial hypertrophy. A substitution of conserved residue, Y237S, in the C1 domain increases calcium sensitivity and accelerates cross-bridge kinetics, also leading to myocardial hypertrophy [57]. Another mutation, E258K, disrupts the interaction of cMyBP-C with the S2 fragment of myosin, which leads to accelerating the kinetics of actin–myosin interaction [76,77,78]. This mutation primarily reduces cMyBP-C expression, which makes haploinsufficiency its principal mechanism of HCM development.

The mutations D75N and P161S studied in our work exhibit molecular mechanisms that are distinct from those of previously investigated amino acid substitutions. Note that the C0-C2 fragments with mutations D75N and P161S disrupted the activation of thin filaments. The P161S mutation, in particular, increased the sliding velocity of thin filaments, which might lead to increased myocardial contractility.

The only other well-studied mutation associated with HCM in the C0 domain, A31P, found in Maine Coon cats, has been shown to disrupt the domain’s structure and stability [79,80]. cMyBP-C with the A31P mutation does not lead to haploinsufficiency. The authors suggested that the pathogenic mechanism of this mutation was associated with impaired cMyBP-C function. We found that the D75N mutation affected the structure of the C0 domain, and our data indicate that the action mechanism of the D75N mutation can be associated with the effect of the C0-C2 cMyBP-C fragment on the myosin S1 function through the interaction of C0 domain with myosin regulatory light chain [81].

## 4. Materials and Methods

### 4.1. Proteins

The porcine heart was acquired from Kamensk PLC, a local farm (Ekaterinburg, Russia). Porcine ventricular myosin and rabbit skeletal actin were prepared by standard methods [82,83]. The human Tpm1.1 (α-Tpm isoform) was expressed in *E. coli* strain C41 (DE3) cells and purified as described previously [84]. Tpm had an Ala-Ser N-terminal extension to mimic the Tpm N-terminal acetylation [85]. The recombinant human cardiac Tn complex (Tn) comprising TnI, TnT, and TnC, expressed in *E. coli*, was provided by HyTest, Turku, Finland (cat. no. 8ITCR).

The complete coding sequence of human cMyBP-C (UniProt Q14896; MYPC3_HUMAN) was synthesized by Evrogen (Moscow, Russia). Using the primers cMYBPC (C0) fw and cMYBPC (C2) rev presented in Table 4, the molecular genetic construct C0-C1-m-C2 (1–455 a.a.) was obtained based on the full sequence. The resulting DNA fragment was cloned into the pet23a+ plasmid between the NdeI and EcoRI restriction endonuclease recognition sites. A His-tag of six histidines and a 3C viral protease recognition site flanked the C-terminal coding sequence of cMyBP-C. The mutant forms D75N and P161S of C0-C1-m-C2 protein were obtained using the Q5 site-directed mutagenesis kit and corresponding primers listed in Table 4. The correctness of the construct’s sequence was verified by Evrogen sequencing.

The recombinant C0-C1-*m*-C2 fragments of wild-type and mutated cMyBP-C were expressed in *E. coli* strain C41 (DE3) cells. Expression was induced by adding isopropyl-β-D-1-thiogalactopyranoside (IPTG) to a final concentration of 1 mM, and cells were cultivated overnight at 30 °C. After expression, cells were collected by centrifugation at 4000× *g* in an Eppendorf 5810R centrifuge (Eppendorf, Hamburg, Germany) for 60 min at 4 °C. The cell pellet was resuspended in chromatography buffer (50 mM Tris-HCl (pH 8.0), 15 mM imidazole, 300 mM NaCl, 1 mM PMSF (phenylmethylsulfonyl fluoride), and 5 mM 2-mercaptoethanol. Cell lysates were frozen at −80 °C until further purification.

When purifying proteins, cell pellets were thawed in chromatography buffer, after which the cell suspension was treated with ultrasound (Sonics & Materials Inc., Newtown, CT, USA) for 10 min. The resulting lysate was centrifuged at 25,000× *g* for 60 min. The supernatant was applied to a HisTrap HP column (Uppsala, Sweden) with a volume of 5 mL and pre-equilibrated with 10 volumes (50 mL) of chromatography buffer. The column was connected to a ProStar 3250 chromatography system (Varian Inc., Mulgrave, Australia) and washed at a speed of 3 mL/min for 20 min. Elution was carried out with an imidazole gradient (15–500 mM) based on chromatography buffer. Fractions containing the highest amounts of protein were pooled and dialyzed against 2 L of 30 mM Hepes-Na (pH 7.3) and 100 mM NaCl overnight. The concentration of the C0-C2 fragments was measured spectrophotometrically using the extinction coefficient Abs0.1% = 0.867; DTT was added to 1 mM and frozen at −80 °C.

### 4.2. Differential Scanning Calorimetry (DSC) Experiments

DSC experiments were performed, as described [86,87], on a MicroCal VP-Capillary differential scanning calorimeter (Malvern Instruments, Northampton, MA, USA) at a heating rate of 1 K/min in 30 mM Hepes-Na buffer, pH 7.3, containing 100 mM NaCl, in the temperature range from 10 °C to 80 °C. The protein concentration was 2 mg/mL. Before DSC experiments, all protein species were reduced by the addition of 5 mM BEE and incubation at room temperature for 1 h. After reduction, proteins were purified on Nap-10 columns (GE Healthcare, Chicago, IL, USA). The reversibility of the heat sorption curves was assessed by reheating the sample immediately after cooling from the previous scan. The thermal unfolding of all the C0-C1-m-C2 fragments was fully irreversible. The temperature dependence of the excess heat capacity was further analyzed and plotted using Origin software v 7.5 (MicroCal Inc., Northampton, MA, USA).

### 4.3. In Vitro Motility Assay

In the experiments, we used the whole myosin. The in vitro motility assay and the filament sliding velocity analysis with the GMimPro2023 software [88] were performed as described previously [19,84]. The experiments were repeated three times with each C0-C2 fragment, and the velocities of 50–100 filaments in each experiment were measured. The sliding velocities of 5–20 filaments whose motion was linear for at least 10 frames were measured.

The mean data of individual experiments were fitted to the following Hill equation: *V* = *V*_max_ × (1 + 10*^h^*^(*p*Ca-*p*Ca50)^)^−1^, where *V* and *V*_max_ are a velocity and the maximal velocity at saturating calcium concentration, respectively; *p*Ca_50_ (i.e., Ca^2+^-sensitivity) is *p*Ca at which half-maximal velocity is achieved, and *h* is the Hill coefficient.

The effect of the C0-C2 fragments on the cross-bridge–cross-bridge cooperativity was studied by the dependence of the sliding velocity of thin filaments on the concentration of myosin loaded into the flow cell. The effects of ATP in concentrations 0–2 mM and ADP in concentrations 0.1–3 mM on the sliding velocity of thin filaments at *p*Ca 4 were studied. A separate cell was loaded for each ATP and ADP concentration.

### 4.4. Binding of cMyBP-C C0-C2 Fragment to F-Actin

To study the ability of the C0-C2 fragment of cMyBP-C to bind F-actin, the C0-C2 fragment (0–2000 nM) was added to the nitrocellulose-coated surface of the flow cell for 2 min; then, the cell was filled with BSA for 5 min and washed three times with AB buffer. Fluorescently labeled F-actin (10 nM) was added for 5 min. The C0-C2 fragment binding to F-actin was measured as the total intensity of the filament images in the flow cell using GMimPro software [88]. The intensity dependence on the fragment concentration had a sigmoidal shape, and the concentrations corresponding to the half-maximal intensity were determined by fitting the pooled data to the Hill equation. For each C0-C2 fragment concentration, at least three flow cells were used, and the intensity was measured in 10 fields of view.

### 4.5. Statistics

Each experiment was repeated three times, and all values were expressed as the mean ± SD. Data analysis was performed using Excel 16 (Microsoft Corp., Redmond, WA, USA) and Origin 8.0 (Origin Lab, Northampton, MA, USA). Statistical significance was estimated using the Student’s *t*-test or Mann–Whitney test.

### 4.6. Molecular Dynamics

The molecular model of C0 and C1 domains of cMyBP-C with short segments of F-actin and Tpm, as suggested by Risi et al. (PDB ID 6CXJ, [20]), was used as a starting structure for the simulations of the D75N substitution. We considered only the second of two resolved C0 domain modes of binding to actin, specifically the one with the 75th residue near the actin surface [20]. Our model included 5 actin monomers, 5 C0 and C1 cMyBP-C domains. Tpm chains were removed from the structure as their side chains were not resolved in the original PDB data.

The effects of P161S mutation in the C1 domain were studied using another atomic model. A more recent structure of C1 cMyBP-C domains with F-actin and Tpm (PDB ID 7TJ7, [65]) was used. Tpm strand with unknown side chains was substituted with the full atomic Tpm structure from the model of the human cardiac actin–tropomyosin–myosin complex (residues 45–210, PDB ID 8EFH, [66]) matching underlying actin monomers using USCF Chimera [89]. Then, a copy of 4 actin monomers with 4 C1 domains in contact with the Tpm strand was matched to the second, Tpm-free long actin helix monomers, so the Tpm strands in the model matched actin helical symmetry.

Four missing residues in the atomic structure of the C1 domain in both models were reconstructed using MODELLER v 10.1 software [89]. The D75N or P161S point substitutions were introduced in the corresponding models with USCF Chimera [90] using a built-in rotamers library [91]. Simulations were performed using the Gromacs v 2021.5 package [92]. AMBER99SB-ILDN force field [93] and TIP3P water model [94] were used for the MD simulations. The unit box borders were at least 1.5 nm away from the protein surface, and Na^+^ and Cl^−^ ions were added to neutralize the charge of the system and set an ionic strength of 0.15 M. Periodic boundary conditions were applied. The 200 ns-long MD runs were performed for each MD model type (one WT and one with point substitution), and the atomic coordinates were saved every 0.2 ns. *H*-bonds and distance analysis were performed using Gromacs built-in functions, and changes in the azimuthal Tpm position on actin were analyzed using homemade software as described [95].

## 5. Conclusions

We investigated the impact of D75N and P161S point mutations in the N-terminal C0 and C1 domains on the structure and regulatory function of the C0-C2 fragment of cMyBP-C to clarify the molecular mechanisms underlying HCM pathogenesis associated with these mutations. Both mutations significantly alter the structure of the C0-C2 fragment, leading to disorganization and a lack of rigidity of the tertiary structure. They also impair the activation of thin filaments and disrupt the calcium regulation of actin–myosin interaction.

Another possible mechanism of cMyBP-C participation in the HCM development is a violation of ATP/ADP exchange in the myosin S1 nucleotide pocket. ADP dose-dependently decreases filament sliding velocity in the presence of WT and P161S fragments. However, in the presence of the D75N fragment, ADP does not affect filament velocity, indicating that the D75N mutation drastically alters nucleotide exchange kinetics by reducing ADP affinity to the ATPase pocket and slowing the myosin cycle. The results suggest that the mechanism of the pathogenicity of the D75N mutation might be related to the impact of the C0-C2 cMyBP-C fragment on myosin S1 function. The results enhance our understanding of the mechanisms of HCM development due to missense mutations in N-terminal domains of cMyBP-C and demonstrate that the D75N and P161S mutations differently affected cardiac contractility.

Therefore, the results of this work show that both studied mutations affect actin–myosin interaction, leading to the worsening of the myocardium contractile function. To correct the contractility, in further clinical trials, the possible use of myosin inhibitors like mavacamten should be considered.

## Figures and Tables

**Table 3:** The effects of cMyBP-C mutations on the ATP binding and ADP release.

C0-C2 Fragment	*c*_ATP_, µM	*c*_ADP_, µM
0 nM C0-C2	164.9 ± 42.5	1414 ± 588
500 nM WT C0-C2	191.4 ± 54.5	766 ± 120
500 nM D75N C0-C2	58.4 ± 12.5 *	-
500 nM P161S C0-C2	197.6 ± 60.5	539 ± 165

*c*_ATP_ and *c*_ADP_ are the ATP and ADP concentrations at which the sliding velocity of thin filaments was half-maximal. The values are expressed as mean ± SD. The * symbol indicates a statistically significant difference between the parameters of the equation in the presence of the C0-C2 fragments with mutations and those of the WT C0-C2 fragment, *p* < 0.05.

**Table 4:** Sequences of the oligonucleotide primers for obtaining the molecular genetic constructs of the C0-C1-m-C2 cMyBP-C fragments.

Primer Name	Primer Sequence (5′ → 3′)
cMYBPC (C0) fw	GAATTCGAGCTCCGTCGACAAGCT
cMYBPC (C2) rev	TCAGTGATGGTGATGGTGATGCGGGCCCTGAAACAGCACTTCCAGGGGCTCTTTCACAAAGAGCTCCGT
cMYBPC D75N fw	TGCCAACCAGGGATCTTACGC
cMYBPC D75N adj	GGGCCCACTTCCCGCAC
cMYBPC P161S fw	GCGGTCACAGGATGGAGAGG
cMYBPC P161S adj	ATCACGAAGAGGCCAATGGG

## Data Availability

The original contributions presented in the study are included in the article/supplementary materials; further inquiries can be directed to the corresponding author.

## Supplementary Materials

The supporting information can be downloaded at https://www.mdpi.com/article/10.3390/ijms252011195/s1.
