# Supplementary material for: The D75N and P161S Mutations in the C0-C2 Fragment of cMyBP-C Associated with Hypertrophic Cardiomyopathy Disturb the Thin Filament Activation, Nucleotide Exchange in Myosin, and Actin–Myosin Interaction
Source: Int J Mol Sci. 2024 Oct 18;25(20):11195. doi: 10.3390/ijms252011195 (PMC11508426; doi:10.3390/ijms252011195)
Supplement: Supplementary file 1 [file ijms-25-11195-s001.zip › ijms-3205211-supplementary.pdf]

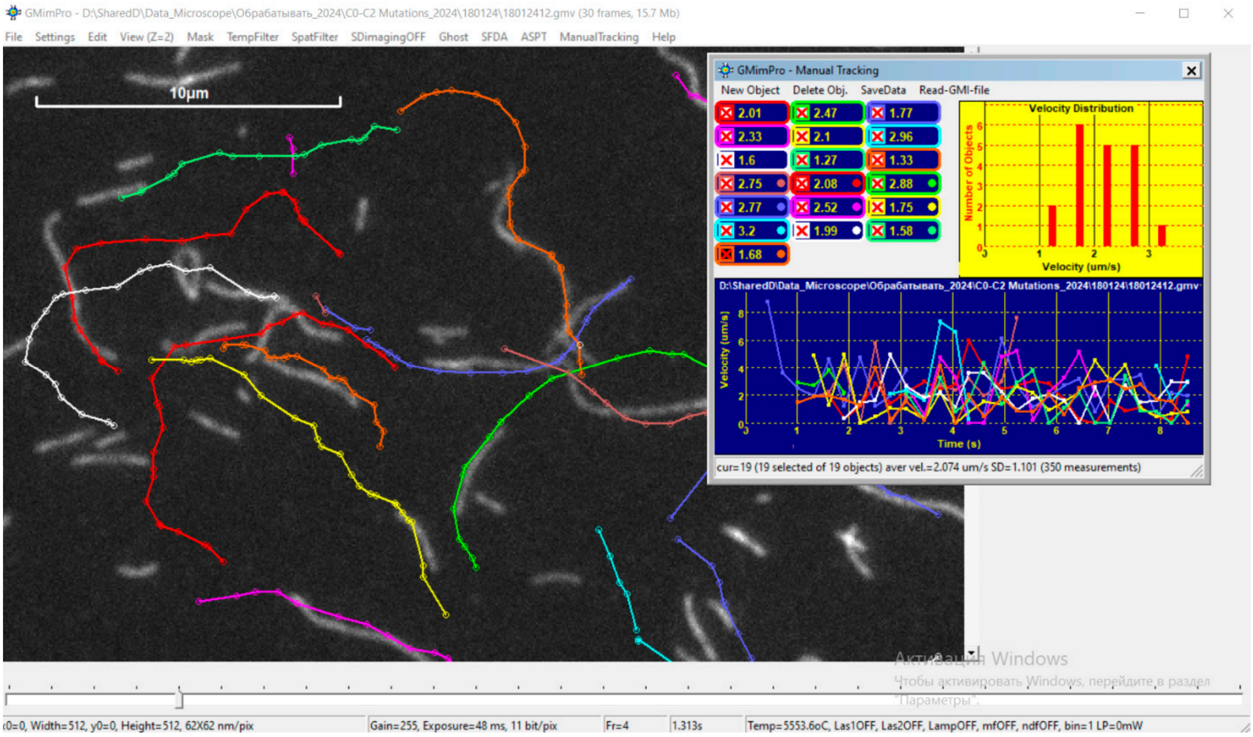

Figure S1. Window of GMimPro software with an example of measuring the sliding velocity of thin filaments at *p*Ca4 without C0-C2 fragment.

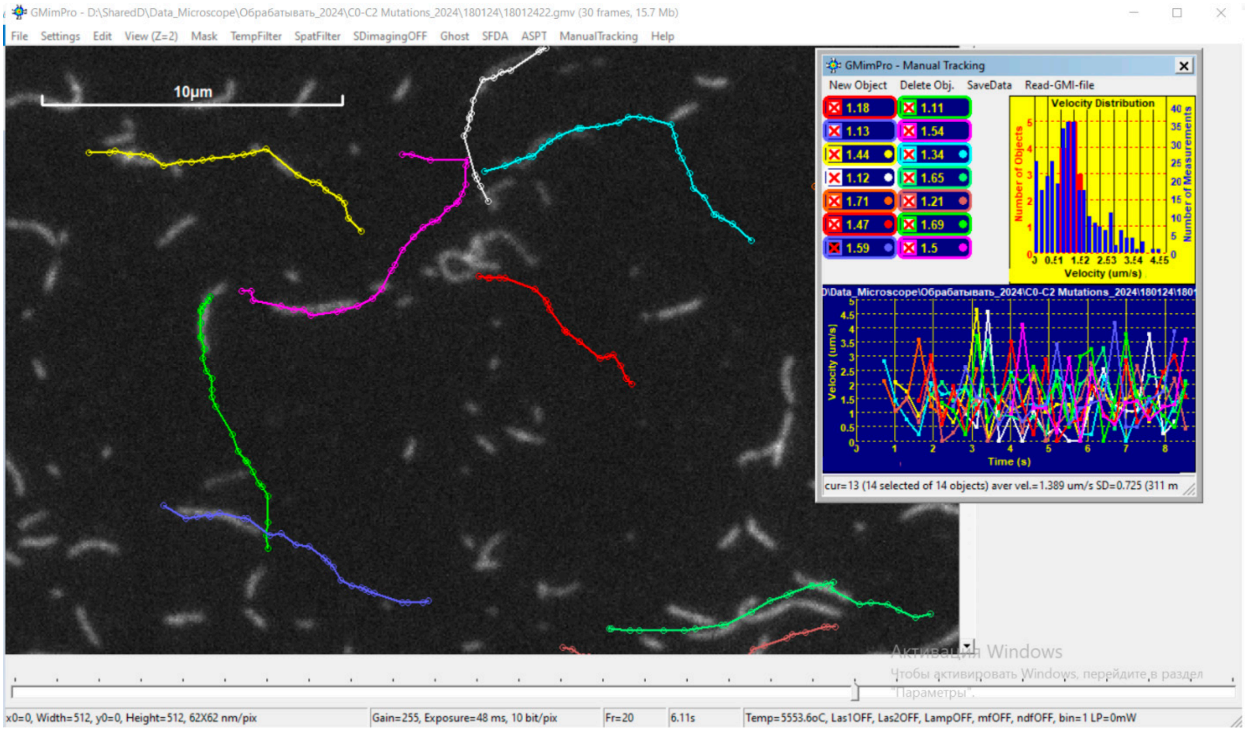

Figure S2. Window of GMimPro software with an example of measuring the sliding velocity of thin filaments at *p*Ca4 with 500nM WT C0-C2 fragment.

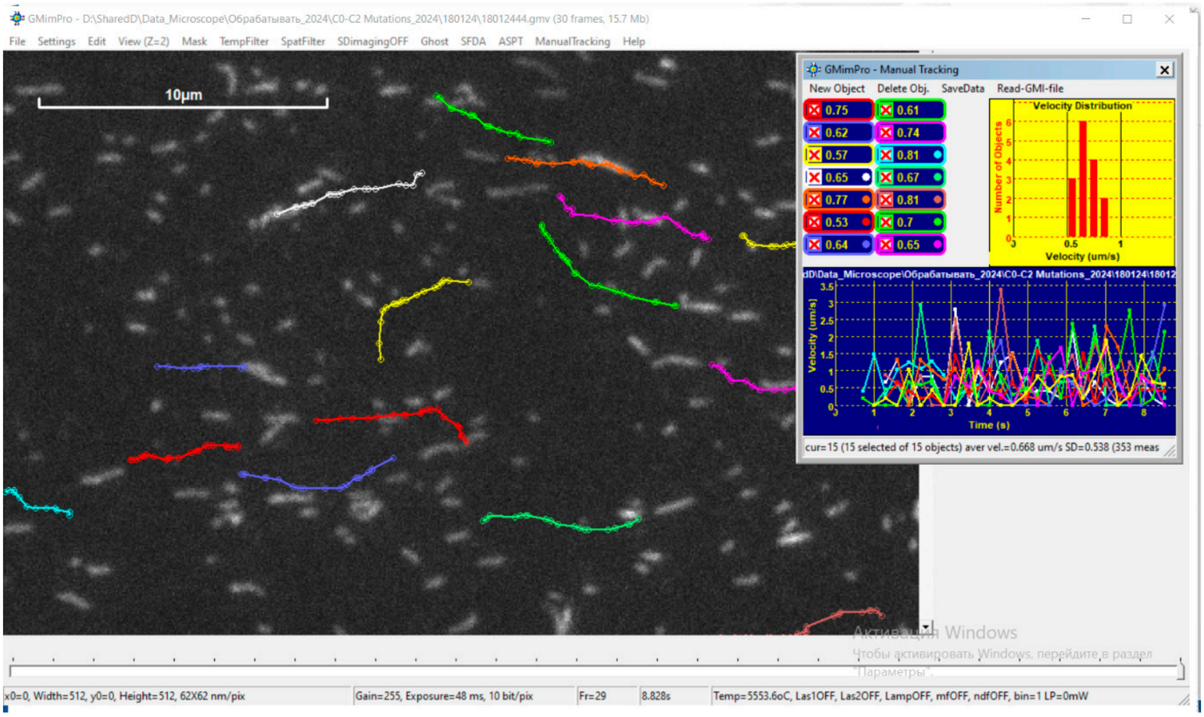

Figure S3. Window of GMimPro software with an example of measuring the sliding velocity of thin filaments at *p*Ca4 with 500nM D75N C0-C2 fragment.

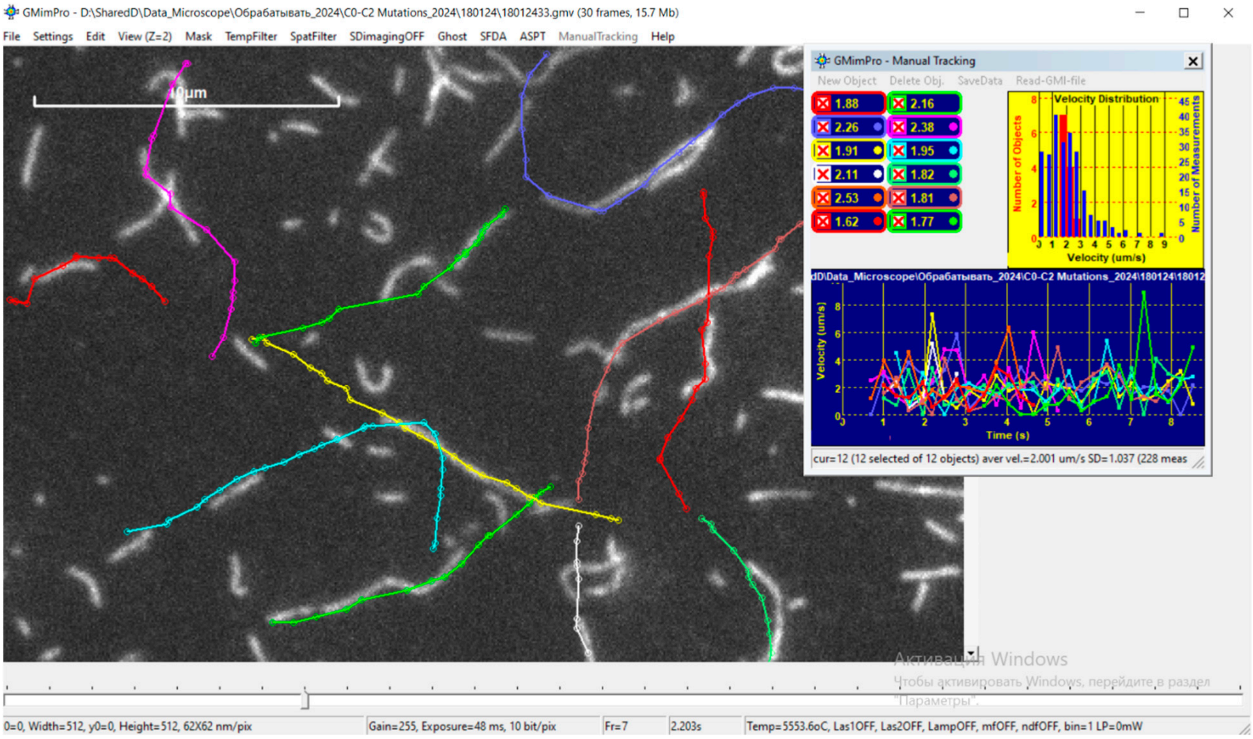

Figure S4. Window of GMimPro software with an example of measuring the sliding velocity of thin filaments at *p*Ca4 with 500nM P161S C0-C2 fragment.

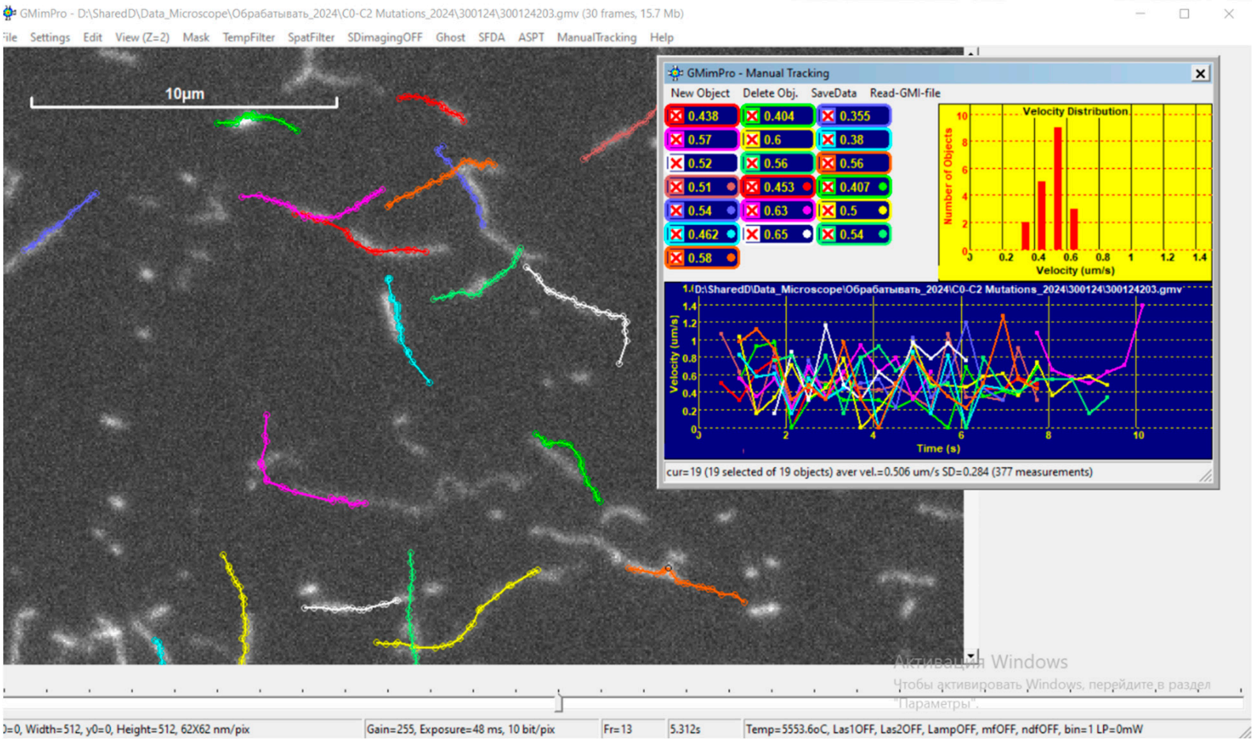

Figure S5. Window of GMimPro software with an example of measuring the sliding velocity of thin filaments in the presence of the D75N C0-C2 fragment at 3000 µM ADP (*p*Ca4).
